# Temporally Regular Musical Primes Facilitate Subsequent Syntax Processing in Children with Specific Language Impairment

**DOI:** 10.3389/fnins.2016.00245

**Published:** 2016-06-20

**Authors:** Nathalie Bedoin, Lucie Brisseau, Pauline Molinier, Didier Roch, Barbara Tillmann

**Affiliations:** ^1^Dynamique Du Langage Laboratory, Centre National de la Recherche Scientifique UMR 5596 and University Lyon 2Lyon, France; ^2^Institut Médico-Educatif FranchemontFranchemont, France; ^3^Lyon Neuroscience Research Center, Auditory Cognition and Psychoacoustics Team, Centre National de la Recherche Scientifique -UMR 5292, INSERM U 1082, University Lyon 1Lyon, France

**Keywords:** SLI, syntax processing, rhythm processing, temporal attention, music

## Abstract

Children with developmental language disorders have been shown to be also impaired in rhythm and meter perception. Temporal processing and its link to language processing can be understood within the dynamic attending theory. An external stimulus can stimulate internal oscillators, which orient attention over time and drive speech signal segmentation to provide benefits for syntax processing, which is impaired in various patient populations. For children with Specific Language Impairment (SLI) and dyslexia, previous research has shown the influence of an external rhythmic stimulation on subsequent language processing by comparing the influence of a temporally regular musical prime to that of a temporally irregular prime. Here we tested whether the observed rhythmic stimulation effect is indeed due to a benefit provided by the regular musical prime (rather than a cost subsequent to the temporally irregular prime). Sixteen children with SLI and 16 age-matched controls listened to either a regular musical prime sequence or an environmental sound scene (without temporal regularities in event occurrence; i.e., referred to as “baseline condition”) followed by grammatically correct and incorrect sentences. They were required to perform grammaticality judgments for each auditorily presented sentence. Results revealed that performance for the grammaticality judgments was better after the regular prime sequences than after the baseline sequences. Our findings are interpreted in the theoretical framework of the dynamic attending theory (Jones, 1976) and the temporal sampling (oscillatory) framework for developmental language disorders (Goswami, 2011). Furthermore, they encourage the use of rhythmic structures (even in non-verbal materials) to boost linguistic structure processing and outline perspectives for rehabilitation.

## Introduction

The role of rhythm in speech processing as well as in language rehabilitation has attracted increased interest (e.g., Fujii and Wan, 2014). Rhythm with its sensorimotor coupling has been proposed to be a powerful stimulator of communication and social interactions, leading to the hypothesis that investigating the relation between rhythm and speech provides relevant insights in the origins of human communication, as well as perspectives for the rehabilitation of neurological disorders, notably by promoting rhythm and music stimulation or training as a potential tool for the rehabilitation of language disorders.

Investigating the potential influence of auditory rhythmic stimulation on language processing has been motivated by links between musical rhythm processing and speech processing for competences as well as deficits. Musical training has been shown to enhance phonological skills (Tierney and Kraus, 2014), even in dyslexic children (Flaugnacco et al., 2015). In typically developing young school-age children, Gordon et al. (2015a,b) showed a strong positive association between rhythm perception skills and expressive grammar skills. Performance in rhythm discrimination tasks predicted grammar skills in children and adults. For example, musical rhythm processing predicted the variance in performance of 6-year-old children for the production of complex syntax and the online reorganization of grammatical information. Furthermore, pre-schoolers with a good capacity to synchronize to the beat score higher on tests of early language skills (e.g., reading readiness), such as phonological processing, auditory short-term memory or rapid naming (Woodruff Carr et al., 2014), in comparison to weak synchronizers scoring low also on the language tests. The link between rhythm and language skills finds further support in data obtained for children with developmental language disorders. Indeed, impaired rhythm and meter processing has been reported in children with Specific Language Impairment (SLI; Weinert, 1992; Corriveau and Goswami, 2009) and in dyslexic children (Overy et al., 2003; Muneaux et al., 2004). SLI children's performance in a paced tapping task (i.e., tapping to a metronome) predicted their performance in word and non-word reading, rime awareness, non-word repetition, and reading comprehension (Corriveau and Goswami, 2009). Similarly, dyslexic children's performance in beat perception predicted word and non-word reading as well as phonological awareness (Muneaux et al., 2004). Congruent findings had been previously reported by Overy et al. (2003) asking dyslexic children to tap to the rhythm of a song (i.e., Happy Birthday), which is a form of syllable segmentation and reflects a type of phonological awareness that is of major importance for acquiring skilled reading.

These rhythm-processing deficits have been suggested to lead to difficulties in accurately processing relevant auditory cues in speech. They can lead to deficits in language perception by disrupting supra-segmental processing required to extract words and syllables from the speech stream (Thomson and Goswami, 2008; Corriveau and Goswami, 2009), and by impairing the to-be-developed phonological representations (e.g., onset-rime awareness), which are also relevant for reading (Muneaux et al., 2004). Impaired encoding of supra-segmental information (e.g., word stress, intonation, rhythm) in SLI and dyslexia has also consequences on syntactic structure processing (Weinert, 1992; Marshall et al., 2009; Sabisch et al., 2009). Syntax deficits are particularly pronounced in SLI, in addition to deficits in phonological and semantic processing (Bishop and Snowling, 2004; Catts et al., 2005).

Rhythmic and temporal processing can be understood in Jones' framework of dynamic attending (e.g., Jones and Boltz, 1989; Jones, 2008). Originally inspired by the processing of musical structures, this framework has been also applied to speech (e.g., Quene and Port, 2005; Kotz et al., 2009). The framework postulates that attention is not equally distributed over time, but develops in cycles: internal oscillators synchronize to the temporal regularities of an external stimulus. They orient attention over time and allow developing expectations about the temporal occurrence of a next event, which then facilitates processing of events at expected time points and facilitates segmentation and structural, temporal integration. Also referring to the dynamic attending theory (Large and Jones, 1999), Goswami (2011) proposed a temporal sampling (oscillatory) framework for developmental dyslexia and, by extension, for SLI. This framework explains phonological impairments and other observed impairments via an underlying deficit in temporal coding and attention.

The use of rhythmic and musical stimulation to improve language processing, and in particular syntax processing, has provided converging evidence for the role of dynamic attending and of internal attentional oscillators for speech processing. The influence of a prior rhythmic stimulation on subsequent syntax processing has been shown for four patient populations who all encounter syntax processing deficits as well as difficulties in temporal processing (including temporal processing in non-verbal materials): patients with basal ganglia lesions (Kotz et al., 2005), patients with Parkinson Disease (Kotz and Gunter, 2015), children with SLI and children with dyslexia (Przybylski et al., 2013).

For patients with basal ganglia lesions who do not show the P600 component evoked by syntactic violations (Kotz et al., 2003), Kotz and colleagues tested whether these patients may benefit from an external, temporally regular stimulation, such as a rhythmically regular (metrical) musical prime. This prime should stimulate internal oscillator set-ups and thus help subsequent speech processing. Patients first listened to a rhythmic prime (i.e., a sequence of a march) for 3 min, followed by the language testing blocks with syntactically correct and incorrect sentences. The external rhythmic stimulation showed a compensatory effect and restored the P600 to syntactic violations in patients with basal ganglia lesions (Kotz et al., 2005) and Parkinson Disease (Kotz and Gunter, 2015).

For children with developmental language disorders (SLI and dyslexia), Przybylski et al. (2013) investigated the potential influence of a musical rhythmic prime on the performance in a subsequent language task requiring syntax processing. They contrasted two musical primes (short musical excerpts played by percussion instruments), for which meter extraction was either easy or difficult (referred to as regular or irregular prime, respectively). In the experimental session, each music presentation was followed by a block of experimental trials of the language task that investigated syntax processing. Children were asked to make grammaticality judgments on auditorily presented sentences that were syntactically either correct or incorrect. Performance of all children (children with SLI, children with dyslexia and control children) in the grammaticality judgments was better after regular prime sequences than after irregular prime sequences. These findings suggest that the rhythmicity of the musical prime can influence temporal attention (e.g., via internal oscillators), which allows reinforcing processes underlying phonological processing, speech segmentation and syntax processing, and that this influence holds over the temporal delay to the language task (i.e., music and language were not presented simultaneously).

However, for these studies, no baseline condition was used for comparison even though a baseline comparison is necessary to judge for the potential benefit of the rhythmic stimulation and its potential perspectives for the development of training and rehabilitation programs. For basal ganglia patients (Kotz et al., 2005), the effect of the musical prime was shown in the restoration of an ERP component (the P600 following the perception of syntactic violations) that was reported as missing in previous work (Kotz et al., 2003). For the developmental language disorders (Przybylski et al., 2013), the effect of the musical prime was shown by comparing two prime types (regular, irregular), thus showing a relative facilitation between the two conditions: regular vs. irregular. However, this comparison does not yet allow concluding about compensatory benefits of the regular prime in comparison to children's performance without music. As in previous linguistic and musical priming research, studying relative facilitation is a first step that then leads to investigating benefits and costs in comparison to a baseline condition, which was the goal of our study.

Building on the previously observed influence of prior music stimulation on subsequent language processing (even though as relative facilitation), our present study aims to investigate the potential benefits provided by a regular musical structure of a preceding sound context on language performance by including the comparison with a baseline condition (e.g., an environmental sound scene that did not include temporal regularities). We focused on the investigation of the potential benefit of the regular prime (and not of the potential cost of the irregular prime) as this result will allow opening for potential avenues in using rhythmic structures (even with non-verbal materials) to boost linguistic structure processing in patient populations. We here tested a group of SLI children and a group of age-matched control children with the experimental set-up of Przybylski et al. (2013), except that we compared performance in syntax processing after a regular musical prime and a neutral baseline condition.

As SLI children have been shown to be impaired not only in rhythm and meter processing (e.g., Corriveau and Goswami, 2009), but also in pitch processing (e.g., (Mengler et al., 2005) for perception; (Clément et al., 2015), for production), we also tested the SLI children's musical abilities for the processing of musical pitch and rhythm, as assessed by two subtests of the abbreviated version of the Montreal Battery of Evaluation of Musical Abilities (MBEMA; Peretz et al., 2013).

## Methods

### Participants

The present experiment included a group of SLI children and a group of control children that were matched for chronological age (CA). For all children, French was the main language, none had benefited from musical training and none reported auditory or visual deficits. For all children, we assessed reading age (RA) with scores obtained with a standardized reading test, the Alouette test, which focuses on decoding mechanisms by requiring children to read sentences without semantic support (Lefavrais, 1965). All children and their parents had given their written informed consent, as well as the director of the institute for the SLI group, prior to the study. The experiment was conducted according to the Helsinki Declaration, Convention of the Council of Europe on Human Rights and Biomedicine, and the experimental paradigm (i.e., a musical prime followed by grammaticality judgments on aurally presented sentences) was approved by the French ethics committee Comité de Protection de Personnes for testing in children with developmental language disorders and typically developing children (see Przybylski et al., 2013).

All SLI children were recruited from the “Institut Médico-Educatif (IME) Franchemont à Champigny sur Marne,” a medical, pedagogical institute with a boarding school for children with language disorders who cannot be accepted in the normal school system. Diagnoses of the language deficit and general neurological assessments were made by neuropsychologists or speech therapists. The evaluations were based on a variety of French neuropsychological and language tests, with pathological scores being defined as scores that were at least two standard deviations inferior to the population mean. The SLI children were not diagnosed as mentally retarded (using WISC IV), even though one child had a score two standard deviations inferior to the population mean in one of the four subtests. All children were not diagnosed with additional learning difficulties (e.g., dyspraxia, ADHD, autistic spectrum disorder or other neurological or psychiatric disorders).

Sixteen SLI children (13 boys, average CA: 9 years 7 months, SD = 13 months, range: 7 years 3 months to 10 years 11 months; average RA: 6 years 11 months, SD = 6 months, range: 6 years 7 month to 8 years 0 month) participated in the experiment. They were diagnosed with a phonological-syntactic syndrome (de Weck and Rosat, 2003) with verbal expression mainly affected at phonological, syntactic, and semantic levels, as assessed by various batteries including at least word and pseudo-word repetition, naming, morphosyntactic production and phonemic fluency. In addition, two children were also diagnosed with receptive dysphasia and one with lexical-syntactic dysphasia. Before the experiment, the SLI children were tested with two further language tests: (1) ECOSSE [“Epreuve de compréhension syntaxico-sémantique” (Test of syntactic-semantic understanding), (Lecoq, 1996)], a French adaptation of the Test for Reception of Grammar (TROG, Bishop, 1983), evaluating the child's syntactic and semantic understanding capacities in spoken language; (2) EVIP [“Echelle de Vocabulaire en Images Peabody” (Scale of Vocabulary evaluated by images)], a French adaptation of the Peabody Picture Vocabulary Test-Revised (Dunn et al., 1993) that uses pictures to assess the child's vocabulary level.

For the ECOSSE, average performance was 76.19 (SD = 23.70; range: 10–110; with 100 being the average score of the reference population). For the EVIP, averaged normalized scores (out of 100) was 91.56 (SD = 16.01; range: 60–117). The children also performed the Raven's Colored Progressive Matrices (Raven et al., 1998) so that we can use their scores to correlate their level of non-verbal intelligence with performance in the syntax task and the MBEMA. The group's average score was 85.53 (SD = 16.31), ranging from 56.5 to 118, with three children performing more than 2 SD below the average score of the reference population (100), but note that none of them had been diagnosed as mentally retarded (see above). See Table 1 for a summarized presentation of the SLI children's performance on these three tests and the reading test (“L'Alouette”). Table 2 presents correlations between results of these four tests and completed with chronological age. The results reveal that none of these features correlated, except for performance between the ECOSSE and the EVIP, both capturing aspects of children's language processing capacities.

**Table 1:** **SLI children's results for the additional neuropsychological tests**.

	**Mean**	**SD**	**Range (min; max)**
ECOSSE	76.19	23.70	10–110
EVIP	91.56	16.01	60–117
Raven's Colored Progressive Matrices	85.53	16.31	56.5–118
Reading age (test “L'Alouette”)	83.31	5.65	78–96

*For ECOSSE, EVIP, and Raven's Progressive Matrices, 100 is the average score of the reference population. For the reading test (“L'Alouette”), we indicate here the scores transformed in reading age, presented in months*.

**Table 2:** **Correlations ***r*** between the SLI children's results in the neuropsychological tests [Raven's Colored Progressive Matrices, Reading test (“L'Alouette,” scores transformed into reading age in months), ECOSSE, EVIPE], the results on the music perception test MBEMA with its pitch and rhythm subtests as well as the patient's chronological age**.

	**CA**	**RA**	**Raven**	**ECOSSE**	**EVIPE**	**MBEMA**	
						**Pitch**	**Rhythm**
Chronological age (CA)	–						
Reading age (RA)	0.30	–					
Raven's Colored Progressive Matrices	−0.24	0.01	–				
ECOSSE	−0.25	−0.16	−0.13	–			
EVIPE	−0.14	−0.45	−0.33	0.63^**^	–		
MBEMA: Pitch	−0.06	−0.27	0.41	−0.37	0.09	–	
MBEMA: Rhythm	0.12	−0.31	0.36	−0.26	0.04	0.61^*^	–

** p < 0.01 (two-tailed);

* *p < 0.05 (two-tailed)*.

Sixteen control children (matched to the SLI children for CA) were included in this study: 9 boys, average CA: 9 years 5 months, SD = 14 months, range: 7 years 4 months to 11 years 0 month; average RA: 9 years 10 months, SD = 17 months, range: 7 years 5 months to 13 years 0 month. None of the children in the control group reported a history of written or spoken language impairments.

### Materials

The regular musical sequence and the linguistic material of Przybylski et al. (2013) were used. Musical and linguistic materials were presented over headphones. The experiment was run on Psyscope software (Cohen et al., 1993).

**Figure 1:**
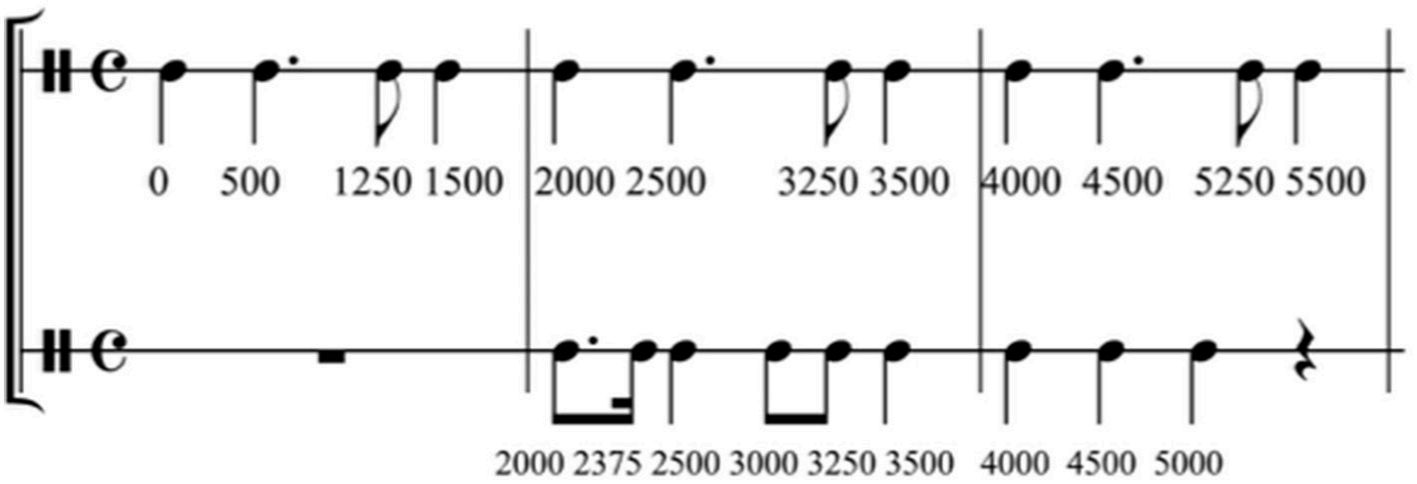
**Musical score of the beginning of the regular musical prime**. The timeline under the score part indicates the onsets of each note (in milliseconds). Adapted from Przybylski et al. (2013), Figure 1.

For the regular prime condition, the musical sequence had a duration of 32 s and contained a rhythmic structure allowing for relatively easy meter extraction (Figure 1; see Supplementary Material). The sequence was played by two percussion instruments (i.e., a tam–tam at 175 Hz and a maracas at 466 Hz), rendering the musical stimulus more attractive than a single line and two voices allowed for reinforcing the underlying beat (e.g., by two simultaneously played events). Each instrumental line was composed of a section of eight beats of 500 ms, which was repeated eight times to form the prime sequence. The simple rhythmic structure consisted of inter-onset-intervals of 250, 500, 750, or 1000 ms and one unit of 375 ms followed by 125 ms (i.e., creating together an interval of 500 ms). To extract the metrical structure, listeners needed to find regular subdivisions of 125 ms, then 250 ms and built a hierarchy with the main beat every 500 ms, followed by another hierarchy level at 1000 ms. The hierarchy was reinforced by the simultaneous presentation of events played by the two instruments on six of the eight beats in the pattern. We selected the tempo of 500 ms based on the developmental work by McAuley et al. (2006) on entrainment; they reported that the spontaneous motor tempo of children of the age from 8 to 10 years lies at about 521 ms (±61).

**Figure 2:**
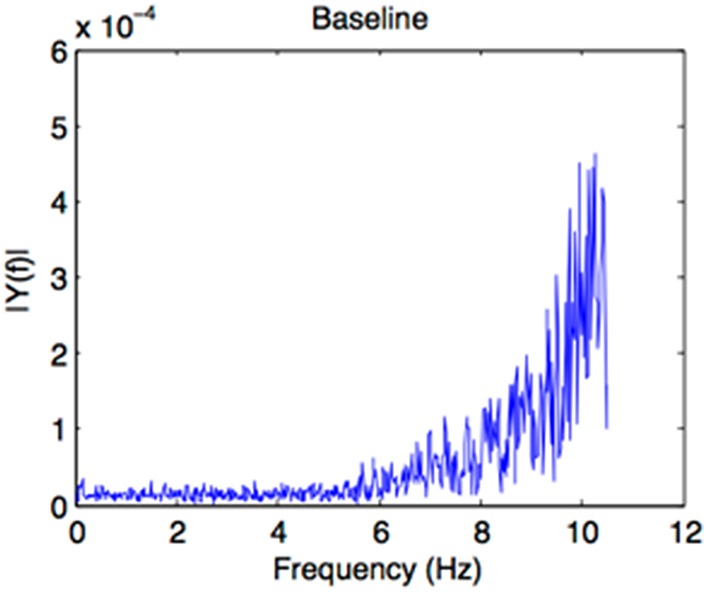
**Result of a Fast Fourrier Transform analysis of the sound file of the baseline prime, confirming that no regularities emerge from the event pattern**.

For the baseline condition, the auditory sequence had a duration of 30 s and presented the recording of an environmental sound scene outside on the street with a playground (Supplementary Material). This environmental scene did not contain temporal regularities in the occurring sounds (as shown by a Fast Fourrier Transform analysis of the sound file, Figure 2) and no comprehensible speech (even though voices were present). The sound file was extracted from the database “universal-soundbank.com.”

The linguistic material was composed of 96 French sentences that were grammatically either correct (48) or incorrect (48). We first created 48 correct sentences, and derived from each correct sentence an incorrect sentence. The violations used were of three different types (Gunter et al., 2000), and affected gender agreement, number agreement or person agreement. Grammatical and ungrammatical sentences were composed of an average of 6.1 words (range of 4 to 8) and an average of 8.29 syllables (range of 6 to 11); their duration was on average 2300 ms (±353). Participants listened to the same sentence in either its grammatically correct version or its incorrect version. For that aim, the 96 sentences were split into two lists (A and B) of 48 sentences. A grammatically correct sentence (presented in list A) was matched in number of words, number of syllables, number of letters and the words' lexical frequency (Lété et al., 2004) with another correct sentence (presented in list B). Based on these lists, two experimental sets were constructed: (1) 24 grammatically correct sentences chosen from list A, and 24 grammatically incorrect sentences from list B (each of the three syntax violation types was represented by eight sentences), (2) 24 grammatically correct sentences chosen from list B, and 24 grammatically incorrect sentences from list A. Each participant worked on one of the sets. Sentences were pronounced by a native female speaker of French with a natural speed of production.

For the MBEMA (abbreviated version, Peretz et al., 2013), we selected the subtests of musical pitch and rhythm. Both tests used 20 unfamiliar tonal melodies (average duration of 3.5 s) that were computer-generated and presented in different musical timbres (e.g., piano, marimba, guitar, flute). Melodies were presented in pairs for 20 trials: 10 trials with identical (same) standard and comparison melodies of a pair and 10 trials with comparison melodies that either differed with a scale-violating tone (pitch subtest) or a change of duration of two adjacent notes (rhythm subtest). For each subtest, there were two additional trials for practice. The material was downloaded from the authors' website^1^. However, at the time of testing, only 17 trials of the pitch subtest (10 different pairs, 7 same pairs) and its practice trials were available and we had to run the test with this reduced version. We transformed performance into percentage of correct response (as in Peretz et al., 2013), but to check whether this reduced version might have influenced performance, we ran an additional group of 16 control children (matched for CA: average age: 9 years 7 months, SD = 13 months, range: 7 years 2 months to 10 years 10 months) aiming to compare performance with that reported in Peretz et al. (2013). For that purpose, we transformed all mean scores into percentages of correct responses. The two subtests of the MBEMA were programmed with Psyscope (Cohen et al., 1993).

### Procedure

The 48 sentences were presented by blocks of six sentences, with the constraint that each block contained three grammatically correct sentences and three incorrect sentences (covering violations of gender, number and person agreement, respectively). Before each of the eight blocks, one of the two prime sequences was presented (with four blocks preceded by a regular prime and four by a baseline prime). The order of the primes and the blocks as well as the order of the sentences in each block were randomized for each participant. Participants were asked to listen to the music and were shown a picture on the computer screen (a black and white drawing, which represented, for example, a guitar playing music). At the end of the prime sequence, a blue exclamation mark appeared on the screen to indicate the beginning of the sentence. Participants were asked to judge the grammaticality of the sentences. To facilitate the understanding of the required grammaticality judgment, it was explained to the children that two dragons pronounced the sentences: one who was never wrong and one who was always wrong. At the end of the sentence, two pictures of dragons were presented on the screen: a dragon who looked satisfied and a dragon who looked puzzled. Participants answered by pressing one of two buttons on the computer keyboard, one below each dragon. The next sentence was triggered by the experimenter. At the beginning, the organization of an experimental trial was illustrated with one grammatically correct sentence and the experimenter performed one trial with the child to make sure that the instructions were understood. While children were listening to the prime sequences, the experimenter listened via headphones to some different music to be unaware of the type of prime sequence presented before the next set of sentences and avoid any unconscious influence on the child's behavior.

For the MBEMA (administered before the main experiment in a separate testing session), we followed the implementation of Peretz et al. (2013): The pitch test was always presented first, followed by the rhythm test, and for each subtest, the order of trials was fixed. Each trial was preceded by a warning tone, followed by the target melody, a 1500 ms silent interval and the comparison melody. Participants were asked to judge for each trial, whether the two melodies were the same or different by pressing one of two response keys on the computer keyboard. The next trial started by pressing a third response key. Note that because of the material availability of the pitch test, we replayed the first three pairs in order to reach 20 test pairs, even though only the first 17 were analyzed.

## Results

### Grammaticality judgments

Performance was analyzed with signal detection theory calculating discrimination sensitivity with *d'* and response bias with *c* for each participant and for each prime condition. These analyses are based on Hits (i.e., correct responses for ungrammatical sentences) and False Alarms FAs (i.e., errors for grammatical sentences) after regular and baseline primes, respectively^2^. *d'* is defined as z(Hits) – z(FAs), and response bias c as −0.5 (z(Hits) ^*^ z(FAs)); see (Macmillan and Creelman, 1991) for more details. *d'* and *c* were analyzed by ANOVAs with prime (regular, baseline) as within-participant factor and group (SLI children, controls) as between-participants factor. To estimate effect sizes, we calculated partial η^2^ (Cohen, 1988).

**Figure 3:**
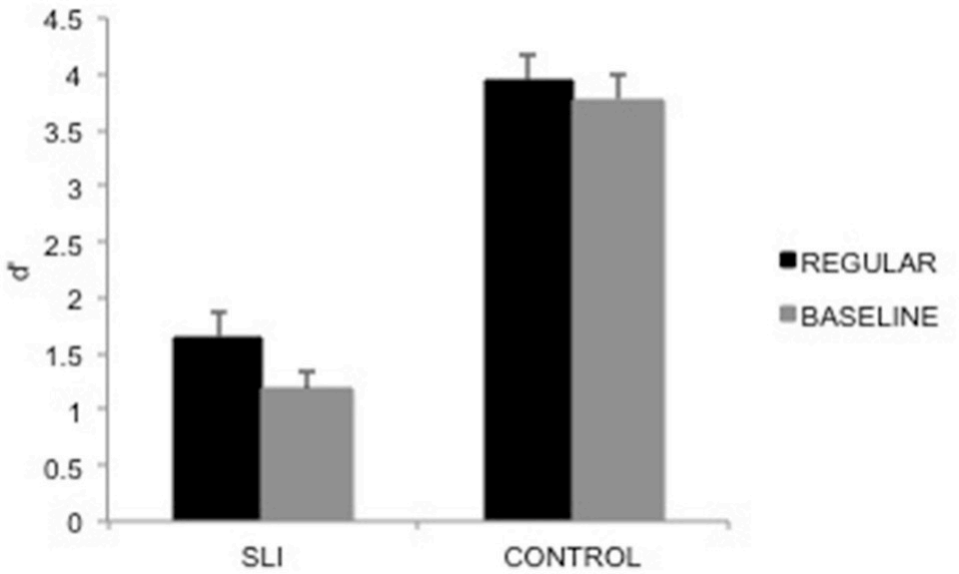
*****d'*** data pattern averaged over participants, presented as a function of the prime (Regular, Baseline), and the participant groups (SLI children, control group)**. Error bars indicate between-participant standard errors.

For *d*' (Figure 3), the main effect of group was significant, *F*_(1, 30)_ = 105.57, *p* < 0.0001, MSE = 0.91, η^2^_*p*_ = 0.78, reflecting, as expected, that controls performed better than SLI children. Most interestingly, the main effect of musical prime was significant, *F*_(1, 30)_ = 4.92, *p* = 0.03, MSE = 0.34, η^2^_*p*_ = 0.14, and it did not interact with group, *p* = 0.32. For all participant groups, performance was better after the regular musical prime than after the irregular musical prime. Average performance suggests that the musical prime effect was reduced in controls due to close-to-ceiling performance. As the focus of the study was on the SLI children, we further checked that the effect for the SLI children was indeed significant when focusing on this participant group, [*F*_(1, 30)_ = 5.24, *p* = 0.03, partial η^2^ = 0.15]. Note that performance for SLI children was above chance level after the regular prime (*p* < 0.0001) and after the irregular prime (*p* < 0.0001). Due to the age range among patients and their CA matched controls, we ran correlational analyses between participants' age and the difference in *d'* for regular and baseline primes; these correlations were not significant over all participants, *r*_(30)_ = 0.12, nor for each participant group considered separately [SLI children: *r*_(14)_ = 0.13; control children: *r*_(14)_ = 0.40].

In addition, we calculated correlations between SLI children's performance (*d'* in the regular condition, *d'* in the baseline condition and the difference in performance between regular and baseline conditions) and their results of the four neuropsychological tests: Raven Matrices (testing for non-verbal intelligence), Alouette test (reading score), ECOSSE (syntactic comprehension) and EVIP (lexical knowledge). Performance in the regular condition correlated with performance of the ECOSSE, *r*_(14)_ = 0.50, *p* < 0.01, and performance in the baseline condition correlated with performance of the EVIP, *r*_(14)_ = 0.66, *p* < 0.01. The other correlations were not significant.

The analysis of *c* (Table 3) revealed that this effect of musical prime was not accompanied by a difference in response bias. Only the main effect of group was significant, *F*_(1, 30)_ = 9.83, *p* = 0.004, MSE = 0.29, η^2^_*p*_ = 0.25, but not the main effect of musical prime, *p* = 0.13, nor their interaction, *p* = 0.89. Note that SLI children showed a bias to respond “grammatical” (with *c* superior to 0) for both the baseline condition (*p* = 0.001) and the regular condition (*p* = 0.01), while control children did not show a response bias that differed significantly from 0, *p*s > 0.68.

**Table 3:** *****c*** data pattern (means, standard errors) averaged over participants, presented as a function of the prime (Regular, Baseline), and the participant groups (SLI children, control children)**.

	**Means**		**Standard errors**	
	**Regular prime**	**Baseline prime**	**Regular prime**	**Baseline prime**
SLI children	0.38	0.42	0.14	0.10
CA controls	0.03	0.01	0.07	0.11

### Pitch and rhythm subtests of the MBEMA

First, we compared the performance of our new control group with performance of children reported in Peretz et al. (2013), notably with the group of 8-year old children [the oldest group tested in Peretz et al. (2013), followed by a group of adult participants]. Performance of the two children groups was highly comparable: Peretz et al.'s children reached 76% of correct responses and 84% for pitch and rhythm subtests, respectively, while our control group here reached 75 and 81% respectively. Based on this, we transformed the cut-off scores of Peretz et al. (2013) into percentages for the pitch subtest (54%) and the rhythm subtest (63%) to evaluate performance of the SLI children. Second, we analyzed performance with a 2 × 2 ANOVA with Group (SLI children, controls) as between-participants factor and test type (pitch, rhythm) as within-participant factor (Table 4). The main effect of group was significant, *F*_(1, 30)_ = 9.29, MSE = 378.84, *p* = 0.005, η^2^_*p*_ = 0.24, with better performance for controls than for SLI children. The main effect of test type was also significant, *F*_(1, 30)_ = 11.08, MSE = 83.25, *p* = 0.002, η^2^_*p*_ = 0.27, with better performance for the rhythm subtest than the pitch subtest (as in Peretz et al., 2013). The interaction between group and test type was not significant, *p* = 0.46. Performance between the two subtests correlated significantly for SLI children [*r*_(14)_ = 0.61, *p* < 0.05] and controls [*r*_(14)_ = 0.71, *p* < 0.01]. As a group, SLI children and control children performed significantly above chance level for the pitch test [SLI: *p* = 0.02; controls: *p* < 0.0001] and the rhythm test [SLI: *p* = 0.0002; controls: *p* < 0.0001]. However, on an individual level, using the cut-off scores from Peretz et al. (2013), more detailed analyses revealed that for the SLI group, eight children were below cut-off for the pitch test and six children for the rhythm test. For the control group, no child was below cut-off for the pitch test, but two children were below cut-off for the rhythm test.

**Table 4:** **Percentages of correct responses (averaged over participants) and standard errors presented as a function of the subtest of the MBEMA (pitch, rhythm) and the participant groups (SLI children, control children)**.

	**Means**		**Standard errors**	
	**Pitch test**	**Rhythm test**	**Pitch test**	**Rhythm test**
SLI children	58.82	68.13	3.56	4.21
CA controls	75.37	81.25	3.09	4.22

*Note that this group of control children was different from that of the main experiment, albeit matched to the SLI group (see text)*.

Based on these findings, we ran supplementary analyses combining the SLI children's results of the MBEMA, our neuropsychological testing and our main experimental task. First, SLI children's performance at the MBEMA did not correlate significantly with their scores at the Raven Matrix [*r*_(14)_ = 0.41 for the pitch test, *r*_(14)_ = 0.36 for the rhythm test], suggesting that their decreased performance cannot be explained by cognitive impairments. Note that it did not correlate neither with the other neuropsychological tests (ECOSSE, EVIPE, reading age) nor chronological age (Table 2). Second, MBEMA performance of either subtest did not correlate significantly with performance in the regular condition [pitch: *r*_(14)_ = 0.07; rhythm: *r*_(14)_ = 0.05], in the baseline condition [pitch: *r*_(14)_ = 0.37; rhythm: *r*_(14)_ = 0.41] or the difference in performance between the two conditions [pitch: *r*_(14)_ = 0.20; rhythm: *r*_(14)_ = 0.25]. Third, the benefit of the regular condition over the baseline condition was comparable for participants performing above or below cut-off for the rhythm test (a difference of 0.41 and 0.57, respectively, *p* = 0.74) and also for the pitch test (a difference of 0.53 and 0.37, respectively, *p* = 0.74). In sum, while SLI children performed below control children in the two subtests of the MBEMA, this decreased performance did not seem to modulate their performance of the main experimental task and the benefit of the regular prime.

## Discussion

The present study builds on previous research having shown the influence of an external rhythmic stimulation on subsequent language processing by comparing the influence of a temporally regular musical prime to that of a temporally irregular prime (Przybylski et al., 2013). We here introduced the necessary baseline condition to investigate whether the observed rhythmic stimulation effect is indeed due to a benefit provided by the regular musical prime. SLI children and their matched controls performed grammaticality judgments after having listened to either a regular musical prime or a rather neutral environmental sound scene. Results revealed better performance (as measured by *d'*) after the regular musical prime than after the baseline prime, and this benefit was not accompanied by a change in response bias (as measured by *c*) between the two conditions. These findings suggest that the previously reported difference between the influence of a temporally regular musical prime and a temporally irregular prime (Przybylski et al., 2013) was not solely due to a cost in processing created by the temporally irregular prime, but included a beneficial effect of the temporally regular prime. We here focused on the investigation of the potential beneficial effect of a temporal regular stimulation as this opens to new perspectives for motivating training and rehabilitation programs. We thus cannot conclude about potentially disturbing effects of the temporal irregular prime on subsequent language processing (i.e., cost of processing in comparison to a neutral baseline condition). However, the comparison of the effect sizes across studies can give us some indication of the potential cost of the irregular prime in addition to the benefit of the regular prime: when comparing regular and irregular primes, the effect size for children with SLI was 0.34 (as measured by partial η^2^; Przybylski et al., 2013), but when comparing regular and baseline primes, the effect size was 0.15 (partial η^2^ reported here). This comparison suggests that the irregular prime also has an influence on language processing, and that thus the observed difference reported by Przybylski et al. (2013) might have included both the benefit of the regular prime and the cost of the irregular prime. However, this cross-study comparison needs to be considered with caution and future studies should directly implement the three experimental conditions (regular, irregular, baseline) in a within-participants design to clearly establish costs and benefits of a temporal context with its irregularities and regularities. In this line, it is worth underlining that determining adequate baseline conditions is difficult (see (Jonides and Mack, 1984), and (Tillmann et al., 2003), for discussions of this difficulty for language and music materials), and it might well be that our baseline prime might have provided some general arousal that would lead to an underestimation of the benefit of the regular prime and an overestimation of the cost of the irregular prime. Future research might want to use a silent prime condition baseline to further study involved benefits, albeit this might be difficult because of the not-yet-known temporal persistence of the musical prime effect over time (thus potentially contaminating a silent baseline condition after having listened to a regular prime condition).

The here observed findings confirm that the previously reported temporal processing deficits in children with developmental language disorders, notably deficits in rhythm and meter processing (e.g., Corriveau and Goswami, 2009), did not hinder the beneficial influence of the regular prime on subsequent language processing, here requiring syntactic processing. Our additional findings on children's capacity of processing pitch and time dimensions (as measured with the MBEMA) are in agreement with this observation. Even though the SLI children performed worse on this test than the control children, their performance levels in the syntax task and their benefit of the regular prime (in comparison to the baseline condition) did not correlate with their performance level in the MBEMA. Even though the MBEMA is not testing for fine-grained temporal processing, this observation suggests that temporal processing capacities should not represent a necessary condition or exclusion criterion to benefit from a rhythmic prime for subsequent language processing. Note that we here observed a deficit in the MBEMA not only in the rhythm subtests (as predicted by previous findings on SLI children's impairments in temporal processing tasks, e.g., Weinert, 1992; Corriveau and Goswami, 2009), but also in the pitch subtest. Both impairments did not correlate with the SLI children's scores at the Raven Matrix test, suggesting that the decreased performance cannot be explained by more general cognitive impairments. Interestingly, the deficit on the pitch dimension is in agreement with other recent findings reporting impaired singing abilities in children with SLI, notably for a pitch-matching task and a melodic reproduction task (Clément et al., 2015). However, even though the SLI group performed below the control group in the pitch and rhythm subtests, the more detailed analyses revealed that on an individual level not all SLI children performed below cut-off (Peretz et al., 2013). These findings thus suggest that verbal and musical deficits can co-occur, but do not necessarily, pointing to the potential heterogeneity of SLI expressions and also indicating that it would be premature concluding for shared deficits in SLI and amusia.

In addition, we also correlated SLI children's performance in the grammaticality task with the results of the neuropsychological tests. While the correlations with Raven Matrices (testing for non-verbal intelligence) and the reading score were not significant, the correlations with the ECOSSE and EVIP tests, measuring syntactic comprehension and lexical knowledge, respectively, were interesting. Participants' performance in the ECOSSE test correlated significantly with participants' performance in the regular prime condition (as measured by *d'*), while participants' performance in the EVIP test correlated significantly with performance in the baseline condition. This finding might be interpreted as an index of stimulation provided by the baseline to help accessing the use of lexical knowledge in the experimental task, while the regular musical prime rather helped to tap into syntactic processing capacities of the children.

Our findings are in agreement with previous research that has shown a beneficial effect of a temporally regular musical stimulus on syntax processing in patients with basal ganglia lesions (Kotz et al., 2005), Parkinson's disease (Kotz and Gunter, 2015) or children with developmental language disorder (Przybylski et al., 2013). For these patient populations, the deficits in temporal processing capacities might affect language processing, which requires sequencing and segmentation (such as syntax here). However, the impaired system can be activated or stimulated by the musical material with its clear metrical structure (clearer than in language material). Indeed, the regular structure provides predictable cues that might boost and entrain internal oscillators, which then benefit the sequencing and temporal segmentation at the sentence level and thus favoring syntax processing. This explanation of the beneficial effect can be tied back to previously proposed hypotheses that SLI children have a sequencing deficit (Weinert, 1992) or a more general procedural deficit (Ullman and Pierpont, 2005). This deficit has been attributed to impaired processing of grammatical structures and temporal sequences—whether language (syntax, morphology, phonology) or music (Ullman, 2001; Ullman and Pierpont, 2005; Corriveau and Goswami, 2009). Together with the previous studies (e.g., Kotz et al., 2003; Przybylski et al., 2013), the findings suggest that non-linguistic stimuli with strongly regular temporal structures might help decreasing this deficit, that is, that they improve cognitive sequencing. As suggested by the dynamic attending theory of Jones (1976), which was then also integrated in the temporal sampling framework proposed by Goswami (2011) to account for impaired rhythmic entrainment (in dyslexia and by extension in SLI), the regular structures entrain internal oscillators and allow guiding temporal attention, thus benefiting for temporal expectancy formation and temporal sequencing more generally. This benefit is particularly relevant for speech processing as speech is tied to time and requires temporal processing and cognitive sequencing (see Kotz and Schwartze, 2010). Kotz et al. (2009) discussed two potential pathways involved in sequencing (i.e., expectancy formation, auditory stream segmentation, syntax processing) and temporal attention: a basal ganglia-preSMA circuitry, and a cerebellar-thalamic-preSMA pathway. These pathways would be involved in the perception of sensory predictable cues (such as beats in metrical structures) and the synchronization between internal oscillators and external (stimulus) regularities (as suggested by the dynamic attending theory, Jones, 1976). Deficits in one of the pathways, such as due to abnormalities in regions of the frontal cortex (in particular Broca's area and pre-motor regions), as reported for SLI (see Ullman and Pierpont, 2005 for a review) might be reduced by stimulating the system with highly regular stimuli (e.g., musical sequences), which may be more efficient for the impaired pathway, or via the alternative pathway, thus allowing for boosting of sequencing capacities and compensating the effect of a sequencing deficit on sentence processing. This has led to the proposition to use musical, rhythmic stimuli and metrical stimulation as a tool for therapeutic interventions or educational practices (e.g., Kotz et al., 2005; Goswami, 2011; Fujii and Wan, 2014), which have been started to be investigated (e.g., Overy, 2000; Flaugnacco et al., 2015). This approach could also exploit the motivational advantages and pleasantness that musical material provides in a training program, beyond its stimulating effect for impaired temporal processing networks.

The importance of sequencing in language processing has also been pointed out by Conway et al. (2009) who focused on the potential origin of the impairment of the involved processes in hearing-impaired listeners. They postulated that sound deprivation in deafness leads to an impaired development of cognitive sequencing capacities affecting speech processing and other structural processing (e.g., they tested the statistical learning of new structured systems). This may be due to the early deprivation from sound in the environment (with its temporal and rhythmic characteristics), which is an efficient source of daily training for cognitive sequencing. Interestingly, deficits in statistical learning have been also reported for SLI children (Evans et al., 2009; Hsu et al., 2014). Together with the dynamic attending theory, this suggests that training of temporal sequencing with auditory non-verbal signals (in particular music with its structural regularities on pitch and time dimensions, leading to expectations about *what* and *when*) could help for speech processing not only in SLI children, but also in hearing-impaired listeners or listeners with cochlear implants. Some recent research has started to use rhythmic primes to improve subsequent language processing in hearing impaired children—either by the immediate repetition of the sentence's accent structure (Cason and Schön, 2012) or on a more abstract level (like in our present study) with a regular musical prime (Bedoin et al., in preparation). For example, Cason and colleagues have shown the effect of a rhythmic prime on subsequent language processing, notably speech perception and speech production (repetition of the presented sentence) in hearing-impaired children (Cason et al., 2015a), as well as performance in a phoneme detection task in the healthy population (Cason et al., 2015b). Most interestingly, Cason et al. have shown that the effect of the rhythmic prime was enhanced when it was associated to movement (participants were required to tap with their hand to the rhythmic structure), thus suggesting the influence of movement and auditory-motor coupling in temporal attention and cognitive sequencing. This finding is also in agreement with the reported beneficial influence of motor activity on the precise temporal encoding of acoustic sequences (Schmidt-Kassow et al., 2013; Chemin et al., 2014; Morillon et al., 2014) as well as the observation of activity in the motor cortex when listening to a temporally regular rhythmic sequence (Fujioka et al., 2012, 2015). These findings thus suggest that it will be interesting to further exploit the association between regular rhythmic stimulation (in particular using music) and movement, aiming to further enhance its beneficial effects for cognitive and temporal sequencing, and in particular speech processing, in rehabilitation settings. Recently, the importance of movement for temporal processing and auditory prediction (predictive timing; temporal expectations) has further been developed in Patel and Iversen's (2014) proposed “Action Simulation for Auditory Prediction” hypothesis, situated in the perspective of evolutionary neuroscience of music perception (and musical beat perception in particular). In this theoretical paper, they propose not only further testable predictions for research investigating the influence of movement on temporal processing, but propose also some speculations about the evolution of beat processing by comparing humans and non-human primates and further motivating cross-species research.

## Author contributions

BT and NB conceived the experiment, analyzed the data and wrote the manuscript. LB, PM, and DR tested participants and analyzed data.

## Funding

This work was conducted in the framework of the LabEx CeLyA (“Centre Lyonnais d'Acoustique”, ANR-10-LABX-0060) operated by the French National Research Agency (ANR).

### Conflict of interest statement

The authors declare that the research was conducted in the absence of any commercial or financial relationships that could be construed as a potential conflict of interest.

## Footnotes

^1^http://www.brams.umontreal.ca/plab/research/Stimuli/mbea_variety_child_version/child_version_mbea_variety_stimuli.html

^2^The correction of the *d'* and *c* measures used .01 for cases without false alarms and .99 for the maximum number of hits.
